# How does weight gain since the age of 18 years affect breast cancer risk in later life? A meta-analysis

**DOI:** 10.1186/s13058-024-01804-x

**Published:** 2024-03-07

**Authors:** Yunan Han, Ebunoluwa E. Otegbeye, Carrie Stoll, Angela Hardi, Graham A. Colditz, Adetunji T. Toriola

**Affiliations:** 1 Division of Public Health Sciences, Department of Surgery, Washington University School of Medicine, 660 South Euclid Avenue, Campus Box 8100, St. Louis, MO 63110 USA; 2 Department of Surgery, Washington University School of Medicine, St. Louis, MO USA; 3 Bernard Becker Medical Library, Washington University School of Medicine, St. Louis, MO USA; 4 Alvin J. Siteman Cancer Center, Barnes-Jewish Hospital and Washington University School of Medicine, St. Louis, MO USA

**Keywords:** Weight gain, Breast cancer, Premenopausal, Postmenopausal

## Abstract

Early life factors are important risk factors for breast cancer. The association between weight gain after age 18 and breast cancer risk is inconsistent across previous epidemiologic studies. To evaluate this association, we conducted a meta-analysis according to PRISMA guidelines and the established inclusion criteria. We performed a comprehensive literature search using Medline (Ovid), Embase, Scopus, Cochrane Library, and ClinicalTrials.gov to identify relevant studies published before June 3, 2022. Two reviewers independently reviewed the articles for final inclusion. Seventeen out of 4,725 unique studies met the selection criteria. The quality of studies was assessed using the Newcastle-Ottawa Scale (NOS), and all were of moderate to high quality with NOS scores ranging from 5 to 8. We included 17 studies (11 case-control, 6 cohort) in final analysis. In case-control studies, weight gain after age 18 was associated with an increased risk of breast cancer (odds ratio [OR] = 1.25; 95% CI = 1.07–1.48), when comparing the highest versus the lowest categories of weight gain. Menopausal status was a source of heterogeneity, with weight gain after age 18 associated with an increased risk of breast cancer in postmenopausal women (OR = 1.53; 95% CI = 1.40–1.68), but not in premenopausal women (OR = 1.01; 95% CI = 0.92–1.12). Additionally, a 5 kg increase in weight was positively associated with postmenopausal breast cancer risk (OR = 1.12; 95%CI = 1.05–1.21) in case-control studies. Findings from cohort studies were identical, with a positive association between weight gain after age 18 and breast cancer incidence in postmenopausal women (relative risk [RR] = 1.30; 95% CI = 1.09–1.36), but not in premenopausal women (RR = 1.06; 95% CI = 0.92–1.22). Weight gain after age 18 is a risk factor for postmenopausal breast cancer, highlighting the importance of weight control from early adulthood to reduce the incidence of postmenopausal breast cancer.

**Supplementary Information:**

The online version contains supplementary material available at 10.1186/s13058-024-01804-x.

## Background

Breast cancer is the most commonly diagnosed cancer among women in the United States [1]. In 2022, it is estimated that approximately 30% of newly diagnosed cancers in women will be breast cancer [1]. Obesity is a modifiable risk factor for breast cancer [2]. However, the relationship between adiposity and breast cancer risk is complex and varies depending on menopausal status. Adiposity before menopause is inversely associated with the risk of premenopausal breast cancer, while adiposity after menopause is positively associated with the risk of postmenopausal breast cancer [3–5].

Long-term weight change can modify breast cancer risk, but the association varies during the life course. Previous studies have produced inconsistent results on the association between weight gain since age 18 and breast cancer risk, as well as whether this association varies by menopausal status [6–9]. Two previous meta-analyses published in 2010 (involving 9 studies) and 2019 (involving 11 studies) used varying early adulthood starting ages (ranging from ages 15 to 25 years old) and did not specifically examine the association between weight gain after age 18 and breast cancer risk [10, 11]. Based on previous studies, we considered that recalled weight at age 18 may be more accurate, as many participants may have entered university at around 18 years old and may have undergone a physical examination for entrance, during which their body weight information would have been recorded [12].

To date, no meta-analysis has provided a pooled estimate specifically on the association between weight gain since the age of 18 years and breast cancer risk in later life. Therefore, an updated meta-analysis is necessary to evaluate this association and determine whether there is heterogeneity by menopausal status. The findings from this study will support public health initiatives aimed at promoting weight control from age 18 onwards to reduce a woman’s risk of breast cancer.

## Methods

### Eligibility criteria

This meta-analysis was conducted according to the Preferred Reporting Items for Systematic Reviews and Meta-analyses (PRISMA) reporting guidelines [13]. This study was registered on PROSPERO (CRD42021091749) and was exempt from institutional review boards as it only included de-identified data found through the public domain in previously published works.

Studies were selected using the “PICOS” format: (1) Population: women aged ≥ 18 years; (2) Intervention: change in weight/body mass index (BMI) from age 18 throughout adulthood; (3) Comparison: not applicable; (4) Outcome: risk of incident breast cancer; (5) Study design: observational studies, including cohort studies, case-control studies, cross-sectional studies, and clinical trials. Case reports, review articles, studies with non-human participants, non-English language articles, and unavailable full-text articles were excluded.

### Search strategy and study selection

The published literature was searched using strategies designed by a medical librarian (A.H., a Master of Library and Information Science [MLIS]-qualified librarian) for the concepts of weight gain or weight change and breast cancer. These strategies were created using a combination of controlled vocabulary terms and keywords and were executed in Medline (Ovid) 1946-, Embase.com 1947-, Scopus 1823-, Cochrane Library (including CENTRAL), and Clinicaltrials.gov. Results were limited to articles in English using database-supplied filters. A filter was also used to exclude animal-only studies from Ovid-Medline and Embase. All searches were completed on June 3, 2022. The full search strategies and terms are detailed in the Supplement. After removing duplicate citations, two reviewers (Y.H. and E.O.) independently screened the titles and abstracts resulting from the medical librarian’s search strategy. Full-text articles were retrieved if they passed the initial screening of title and abstract. Based on the pre-specified selection criteria, both authors independently reviewed the full-text articles for final inclusion. Disagreements were resolved via discussion.

### Data extraction

Two independent reviewers (Y.H. and E.O.) extracted the required data from eligible studies using an author-created extraction form. The primary outcome measures were relative risks (RRs)/hazard ratios (HRs) in cohort studies or odds ratios (ORs) in case-control studies along with the corresponding 95% confidence intervals (CIs). We extracted the adjusted risk estimates (REs) (e.g., RRs, HRs, or ORs) and 95% CIs reflecting the greatest degree of adjustment for possible confounding factors from regression models. We defined a change in weight or body mass index (BMI) as a change in weight (in pounds or kilograms) or BMI (weight in kilograms divided by height in meters squared) as measured from age 18 to the date of the breast cancer diagnosis (for case-control studies) or the last follow-up before diagnosis or until inclusion (for cohort studies). For the meta-analysis, we used the adjusted REs with 95% CIs for the largest weight gain group compared to the reference group from each study. In instances where some studies reported more than one RE for stratified groups, we took separate REs. We also extracted data on weight change in continuous form. In case-control studies, we recorded the number of cases and controls, while in cohort studies, we reported the total cohort number and breast cancer incident cases. We also extracted data on menopausal status (pre-, or postmenopausal), race/ethnicity, tumor hormone receptor status (ER, PR, HER2), along with details about weight change such as categorical vs. continuous. Other data of interest included the study’s detailed information such as the first author’s last name, year of publication, country (U.S.A. or other countries), study design (case-control or cohort), data source, measures of effect size, and factors adjusted for in the model.

### Assessment of bias risk

Two authors (Y.H. and E.O.) independently used the Newcastle-Ottawa Scale (NOS) to assess the quality of each study based on selection, comparability, and exposure (in case-control studies) or outcome (in cohort studies) [14]. The NOS assigns a maximum sum score of 9 for both case-control and cohort studies, with higher scores indicating higher study quality. In addition, we created a three-category scoring system to evaluate study quality, including reporting of enrollment dates, funding sources, and conflicts of interest (Table 1). Quality assessments were compared between the two reviewers, and any disputes were resolved through discussion. We assessed heterogeneity among study-specific estimates using the chi-squared (Cochran Q statistic) and I^2^ statistic. I^2^ values less than 25% were considered low heterogeneity; I^2^ values between 25% and 50% were considered moderate heterogeneity; I^2^ values greater than 50% were considered high heterogeneity [15, 16]. We also assessed for publication bias using funnel plot asymmetry and the Egger test [17, 18].

**Table 1:** Characteristics of studies meeting search inclusion criteria

Author	Year	Country	Participants	Study design	Data source	Menopausal status	Participant number	Adiposity change information	Breast cancer incidence by hormone receptor status	Breast cancer incidence by race/ethnicity	NOS score	Study quality			Measures of effect size	Factors adjusted for in the model
												Reported Enrollment Dates	Reported funding source	Reported conflicts of interest		
Cao [21]	2019	China	Asian (Chinese)	Case-control	The Chinese Wuxi Exposure and Breast Cancer Study	Premenopausal	Cases: 254 Controls: 362	Weight (kg); Categorical (gain of ≤ 0, 0-5.6 [Ref], 5.7–9.5, 9.6–14, > 14); Continuous: per 5 kg weight change	NA	NA	7	Yes	Yes	Yes	Odds ratio (OR)	Age, education, age at menarche, age at first birth, parity, age at menopause, family history of breast cancer, previous benign breast disease, use of hormone replacement therapy, use of oral contraceptives, alcohol consumption, physical activity, height, weight at age 18.
						Postmenopausal	Cases: 518 Controls: 517									
Rosner [22]	2017	U.S.A.	Americans	Prospective cohort	The Nurses’ Health Study cohort	Premenopausal	Cohort: 6,894 Breast cancer cases: 758	Weight (kg); Categorical (loss of > 5, no change ≥-5, ≤ 5 [ref], gain of 5.1–10, 10.1–14.9, 15-19.9, ≥ 20); Continuous: per 30 kg weight change	ER+/PR+; ER+/PR-; ER-/PR-	NA	6	Yes	Yes	No	Hazard ratio (HR); log-incidence model	(i) duration of premenopause, (ii) duration postmenopause, (iii) type of menopause, natural or surgical (iv) parity and age at each birth, (v) current, past hormone therapy (HT) use, (vi) duration of HT use by type (estrogen only vs. estrogen plus progestin E&P), (vii) adult height, (viii) benign breast disease (BBD), (ix) alcohol intake, (x) family history of breast cancer in a first degree relative.
						Postmenopausal	Cohort: 44,691 Breast cancer cases: 4,207									
Wu [23]	2016	U.S.A.	Asian Americans (Chinese, Japanese, Filipina)	Case-control	Los Angeles County Cancer Surveillance Program	Premenopausal	Cases: 937 Controls: 1,025	Weight (kg); Categorical (gain of ≤ 3.64, 3.64–9.09 [ref], 9.09–14.1, 14.1–22.7, > 22.7); Continuous: per 5 kg weight change	NA	NA	5	Yes	Yes	Yes	OR	Age, education, income, years of residence in the United States among non-U.S. born, interviewer, age at menarche, parity, family history of breast cancer, and benign breast diseases, Asian ethnicity.
						Postmenopausal	Cases: 1,133 Controls: 882									Age, education, income, years of residence in the United States among non-U.S. born, interviewer, age at menarche, parity, family history of breast cancer, benign breast disease, and type of menopause status and age at menopause, Asian ethnicity.
Iqbal [24]	2015	Bangladesh	Asian (Bangledeshis)	Case-control	Four hospitals in Bangladesh	Premenopausal	Cases: 129 Controls: 129	Weight (kg); Continuous: per unit kg weight gain	NA	NA	6	Yes	Yes	Yes	OR	Reproductive, anthropometric, and socioeconomic factors.
Robinson [6]	2014	U.S.A.	Black Americans, White Americans	Case-control	Carolina Breast Cancer Study	Premenopausal	Cases: 848 Controls: 685	Weight (Ibs); Categorical (gain of ≤ 25 [ref], 26–54, ≥ 55)	ER + or PR+; ER- and PR-	Yes (Black Americans, White Americans)	5	Yes	Yes	Yes	OR	Age, age squared, family hx, alcohol, menarche, parity and age at 1st FTP composite, lactation, education, smoking, reference BMI.
						Postmenopausal	Cases: 899 Controls: 818									
Kawai [25]	2014	U.S.A.	Americans	Case-control	Three-county Seattle-Puget Sound metropolitan area (King, Pierce, and Snohomish counties)	Premenopausal	Cases: 1021 Controls: 940	BMI (kg/m^2^); Categorical (gain of < 0, 0–5 [ref], 5–10, ≥ 10); Continuous: per unit BMI change	ER-/PR-/ HER2-; ER-/ HER2+; ER+	NA	6	Yes	Yes	Yes	OR	Age at reference, reference year, race/ethnicity, and age at first birth.
Canchola [7]	2012	U.S.A.	Americans	Prospective cohort	California Teachers Study cohort	Postmenopausal	Cohort: 52,642 Breast cancer cases: 2,839	Weight (Ibs); Categorical (loss of ≥ 10, stable≥-10, ≤ 10 [ref], gain of10-24, 25–39, ≥ 40); Continuous: per 10 lbs weight gain	ER+/PR+; ER+/PR-; ER-/PR-	NA	7	Yes	Yes	Yes	Relative risks (RR; hazard rate ratios); Cox proportional hazards regression models	For ER+/PR+: age as the timescale, were stratified by age at baseline, and adjusted for age at menarche, parity, age at first full-term pregnancy, history of benign breast biopsy, family history of breast cancer, alcohol consumption, and use of hormone therapy, height, height. For other subtypes of breast cancer, please see the original paper.
Ahn [8]	2007	U.S.A.	Americans	Prospective cohort	National Institutes of Health -American Association of Retired Persons (NIH-AARP) Diet and Health Study	Postmenopausal	Cohort: 99,039 Breast cancer cases: 2,111	Weight (kg); Categorical (gain of≥-7, -6.99 to -2, -1.9-1.9 [ref], 2-9.9, 10-19.9, 20-29.9, 30-39.9, 40-49.9, ≥ 50)	NA	NA	6	Yes	Yes	Yes	RR; Cox proportional hazards regression	Age, age at menarche, age at menopause, age at first birth, parity, smoking, educational level, race, family history of breast cancer, fat intake, alcohol consumption, oophorectomy, and physical activity, BMI at age 18 years, current BMI.
Palmer [43]	2007	U.S.A.	Americans	Prospective cohort	The Black Women’s Health Study	Premenopausal	Cohort: 42,538 Breast cancer cases: 490	Weight (kg); Categorical (gain of < 10 [ref], 10–14, 15–19, 20–24, ≥ 25)	ER+/PR+; ER+/PR- or ER-/PR+; ER-/PR-	Yes (Black Americans)	6	Yes	Yes	No	RR; Cox proportional hazards regression models	Age, age at menarche, parity, age at first birth, vigorous activity, education, and family history of breast cancer, BMI at age 18 y.
						Postmenopausal	Cohort: 9,542 Breast cancer cases: 443	Weight (kg); Categorical (gain of < 10 [ref], 10–14, 15–19, 20–24, ≥ 25)								Age, age at menarche, parity, age at first birth, age at menopause, vigorous activity, education, and family history of breast cancer, BMI at age 18 y.
Weiderpass [26]	2004	Norway and Sweden	Norwegian and Swedish	Prospective cohort	Central Population Register	Premenopausal	Cohort: 99,717 Breast cancer cases: 733	BMI (kg/m^2^); Categorical (gain of < 0, 0-1.4 [ref], 1.5-4, > 4)	NA	NA	8	Yes	Yes	No	RR; Cox proportional hazard models	Age at enrolment, parity, age at first birth, oral contraceptive use, age at menarche, family history of breast cancer, total duration of breast-feeding, and country of residence, BMI at enrolment.
Wenten [27]	2002	U.S.A.	Non-Hispanic White Americans, Hispanics	Case-control	New Mexico Women’s Health Study	Premenopausal	Cases: 221 Controls: 314	Weight (kg); Categorical (gain of < 4 [ref], 4–7, 8–14, >14)	ER+/PR+; ER-/PR-	Yes (Non-Hispanic White Americans, Hispanics)	5	Yes	Yes	No	OR	Age, family history of breast cancer (1st degree relative), total METS, parity, oral contraceptive use, months of breast feeding, age at first full-term birth, and weight at 18.
						Postmenopausal	Cases: 349 Controls: 391									Age, family history of breast cancer (1st degree relative), total METS, parity, oral contraceptive use, months of breast feeding, age at first full-term birth, HRT use, and weight at 18.
Morimoto [9]	2002	U.S.A.	Americans	Prospective cohort	The women’s health initiative	Postmenopausal	Cohort: 85,917 Breast cancer cases: 1,003	BMI (kg/m^2^); Categorical (gain of < 0, 0-3.5, 3.5–6.2, 6.2–9.7, > 9.7)	NA	NA	7	Yes	Yes	No	RR; Cox proportional hazards regression	Age, education, age at menopause, parity, age at first birth, first degree family history of breast cancer, smoking, age at menarche, race, alcohol consumption, recreational physical activity, dietary energy.
Li [28]	2000	U.S.A.	Americans	Case-control	Thirteen counties in western Washington State and participates in the Surveillance, Epidemiology and End Results Program (SEER) of the National Cancer Institute	Postmenopausal	Cases: 478 Controls: 433	Weight (Ibs); Categorical (gain of < -10, -10 to 10 [ref], 11–30, 31–50, 51–70, > 70)	NA	NA	6	Yes	Yes	No	OR	Age, height, weight at age 18, family history of breast cancer, parity, use of hormone replacement therapy, and oral contraceptive use.
Magnusson [29]	1998	Sweden	Swedish	Case-control	Swedish regional cancer registries	Postmenopausal	Cases: 2,331 Controls: 2,214	Weight (kg); Categorical (gain of < 0, 0-9.5 [ref], 10-19.5, 20-29.5, ≥ 30)	NA	NA	7	Yes	Yes	No	OR	Age parity, age at first birth, age at menopause and use ofhormone replacement therapy.
Trentham-Dietz [30]	1997	U.S.A.	Americans	Case-control	Wisconsin, Massachusetts (excluding the four counties that comprise metropolitan Boston), Maine, and New Hampshire	Premenopausal	Cases: 1,608 Controls: 2,710	Weight (kg); Categorical (gain of < 0, 0-3.1 [ref], 3.2–6.7, 6.8–13.5, 13.6–78); Continuous: per 5 kg weight change	NA	NA	7	Yes	Yes	No	OR	Parity, age at first full-term pregnancy, age at menarche, family history of breast cancer, recent alcohol consumption, education, and height, weight at age 18 years.
						Postmenopausal	Cases: 4,807 Controls: 6,134	Weight (kg); Categorical (gain of < 0, 0-5.8 [ref], 5.9–11.2, 11.3–18, 18.1–93); Continuous: per 5 kg weight change								Parity, age at first full-term pregnancy, age at menarche, family history of breast cancer, recent alcohol consumption, education, age at menopause, and height, weight at age 18 years.
Taioli [31]	1995	U.S.A.	Americans	Case-control	NA	Premenopausal	Cases: 196 Controls: 191	Weight (kg); Categorical (gain of ≤ 0 [ref],1-9.9, 10-19.9, ≥ 20)	NA	NA	6	Yes	No	No	OR	Age, education, age at menarche, pregnancies, physical activity at the age 15–22 years.
						Postmenopausal	Cases: 421 Controls: 340									
Folsom [32]	1990	U.S.A.	Americans	Case-control	State of Iowa	Postmenopausal	Cases: 229 Controls: 1,839	Weight (kg); Categorical (gain of < 8.2, 8.2–17.3, > 17.3)	NA	NA	7	Yes	Yes	No	OR	Age

### Statistical analysis

We performed a meta-analysis on qualifying studies that reported adjusted REs with the corresponding 95% CIs for the association between weight gain from age 18 and breast cancer incidence. A summary of REs with 95% CIs was calculated using a random-effects method, which accounts for possible variations of associations across the studies. We separated the meta-analysis into two groups: (1) case-control studies and (2) cohort studies to allow analysis for RR and OR separately [19]. Case–control estimates were presented as ORs with 95% CIs, while cohort estimates were presented as RRs with 95% CIs. We conducted planned subgroup analyses based on country of study (U.S.A. vs. other countries), menopausal status (premenopausal vs. postmenopausal), and hormone receptor status (positive vs. negative). Additionally, we conducted a separate meta-analysis for studies using continuous weight gain (per 5 kg) as a measurement. We also conducted sensitivity analyses using the “one-out” method, where one study is excluded at a time, and the impact of removing each study is evaluated on summary results and between-study heterogeneity [20]. We specifically considered excluding the cohort study by Rosner (2017), which reported HRs in the meta-analysis for cohort studies. All statistical analyses were performed with STATA version 17 (StataCorp LLC, College Station, TX). All *P* values were two-tailed, and the significance level was set at 0.05.

## Results

### Study selection

**Fig. 1:**
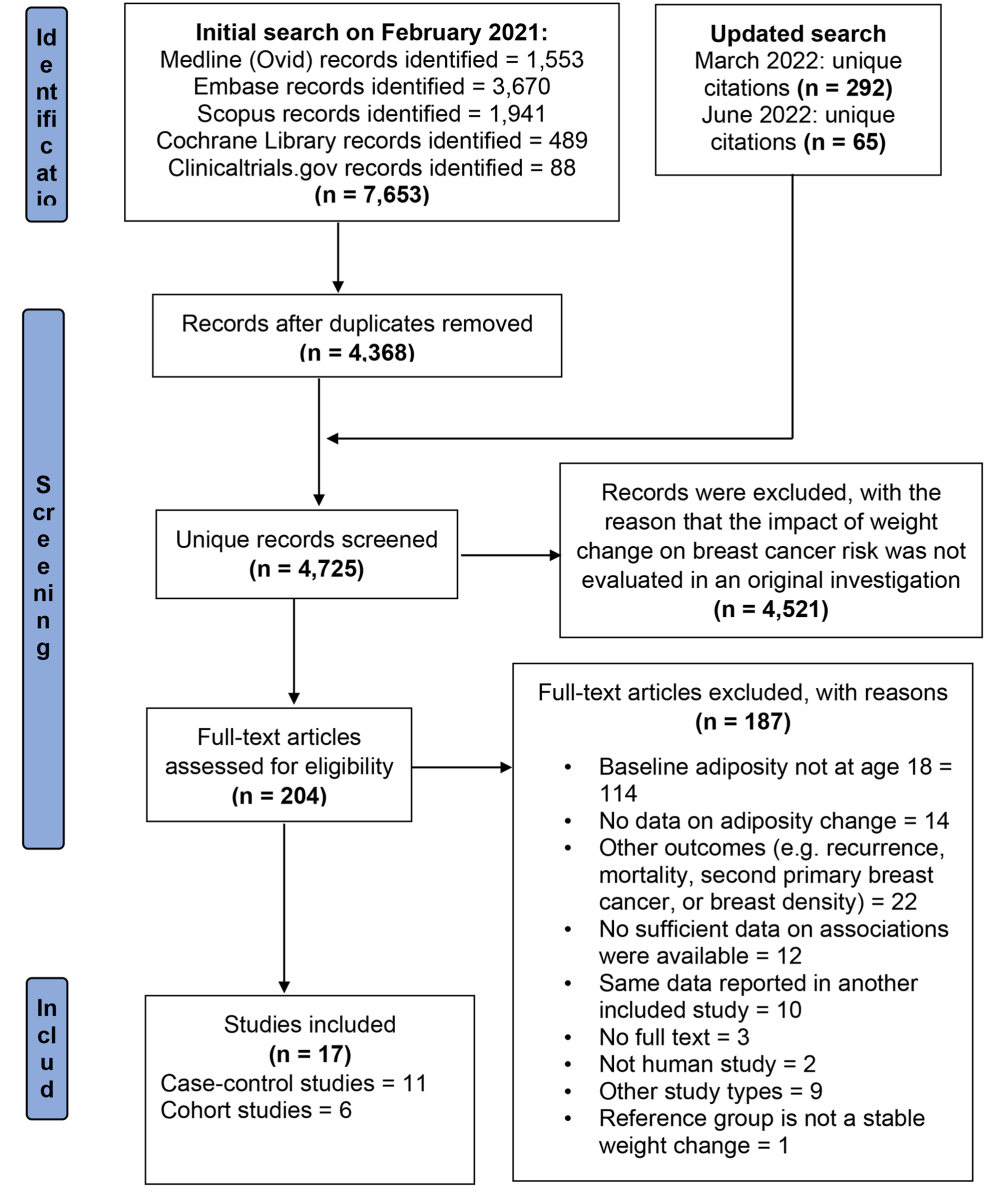
PRISMA flow diagram of systematic literature search

We present the PRISMA flow diagram of our systematic literature review in Fig. 1 [13]. Our initial search in February 2021 yielded 7,653 articles. After removing duplicates, we were left with 4,368 records. We performed two additional updates to the search, adding 292 unique citations in March 2022 and 65 unique citations in June 2022. In total, we screened 4,725 unique articles. After reviewing the titles and abstracts, we excluded 4,521 articles that did not meet the evaluation criteria for the relationship between weight change and breast cancer risk. We then carefully reviewed the full text of the remaining 204 articles and assessed their reference lists for relevant publications, but we did not retrieve any additional studies that met our inclusion criteria. After a thorough review, we excluded 187 publications for not adhering to our inclusion criteria, resulting in a final selection of 17 studies that met our inclusion criteria [6–9, 21–32].

### Study characteristics

We summarized the characteristics of the 17 studies (11 case-control studies and 6 cohort studies) in Table 1. The studies were published between 1990 and 2019. Thirteen studies were conducted in the United States, while the remaining 4 studies were carried out in Bangladesh, China, Norway, and Sweden. All studies reported menopausal status and the number of participants. Six studies stratified their results by breast tumor receptor subtypes, and only three studies provided information on the race/ethnicity of participants. Weight/BMI change was categorized in all studies (14 studies measured in weight, and 3 studies measured in BMI). Additionally, three studies also reported results using continuous weight change (per 5 kg weight). All studies adjusted for age. All 11 case-control studies reported ORs, 5 out of 6 cohort studies reported RRs, and Dr. Rosner’s cohort used HRs as the effect size measure [22].

### Study quality

Quality assessment was performed using a 3-category scoring system and the NOS scores. Based on the 3-category scoring system, seven studies are of high quality (score 3 out of 3), nine studies were of medium quality (score 2 out of 3), and one study was of low quality (score 1 out of 3) (Table 1). All 17 studies reported the participants’ enrollment dates, 16 studies reported the funding sources, and 7 studies declared no conflicts of interest (Table 1). NOS is specifically used for nonrandomized studies and has been endorsed by the Cochrane collaboration. We used the version for case-control studies or cohort studies as applicable, addressing subject selection, study comparability, and the assessment of outcome or exposure. NOS scores ranged from 5 to 8 (9 being the highest possible score), with a mean of 6.3, a median of 6, and a mode of 6 (Table 1). Further details of the NOS scores are shown in Supplementary Tables 1 and 2. All studies received a star for comparability with respect to age adjustment. Except for one study, all studies received an additional star for comparability as they also adjusted for at least one additional risk factor for breast cancer, such as age at menarche, age at first birth, family history of breast cancer, use of hormone replacement therapy, alcohol consumption, or weight at age 18.

### Small-study effects and publication bias

We assessed publication bias and small-study effects using standard funnel plot and Egger regression-based statistical tests. The funnel plots for both case-control and cohort studies were symmetric, indicating the absence of publication bias (Supplementary Figs. 1 and 2). The Egger regression-based statistical tests (all *P*-values > 0.05) also showed no significant evidence of asymmetry in the funnel plots. Moreover, sensitivity analyses demonstrated that any potential publication bias had minimal impact on the overall results.

### Meta-analysis

#### Case-control studies

We included 11 case-control studies with 21 separate ORs in the meta-analysis (Supplementary Fig. 3). When comparing the highest versus the lowest categories of weight gain, we found a significant association between weight gain after age 18 and breast cancer incidence with an OR of 1.25 (95% CI, 1.07–1.48) (Supplementary Fig. 3).

**Fig. 2:**
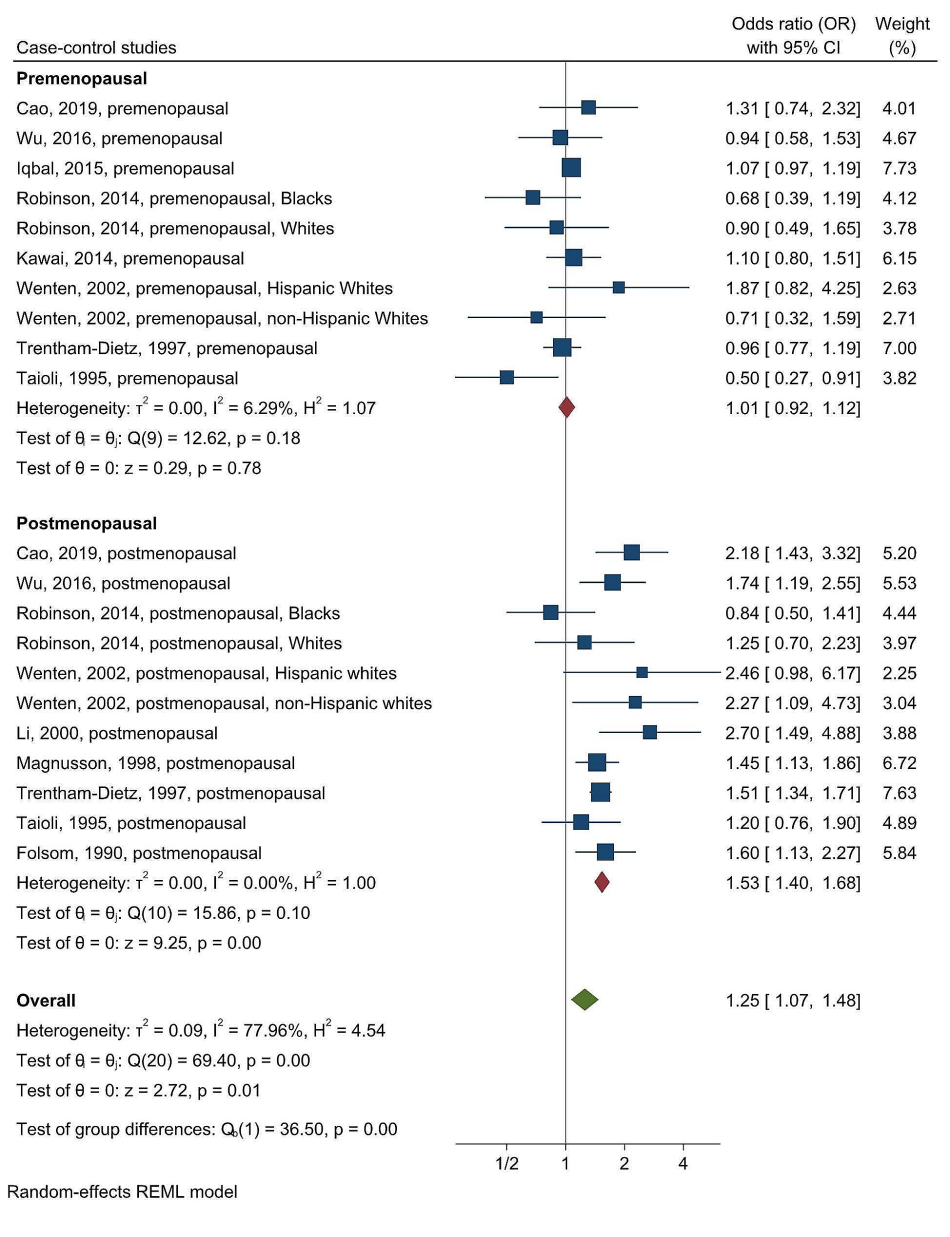
Forest plot for the association between weight gain after age 18 and breast cancer risk in case-control studies, stratified by menopausal status

Menopausal status was a source of heterogeneity (chi-squared test statistic of 36.5 and a *p*-value < 0.001; Fig. 2). When comparing the highest versus the lowest categories of weight gain, the OR decreased to 1.01 (95% CI, 0.92–1.12), and the I^2^ value decreased to 6.29% in premenopausal women; the OR increased to 1.53 (95% CI, 1.40–1.68), and the I^2^ value decrease to 0% in postmenopausal women (Fig. 2). These results underscore a significant association between weight gain after age 18 and breast cancer incidence in postmenopausal, while such an association was not observed in premenopausal women. This highlights that menopausal status is a strong driver of heterogeneity. Furthermore, the 95% CIs did not overlap between the pre- and postmenopausal women, which also suggests a difference in effect size between them.

**Fig. 3:**
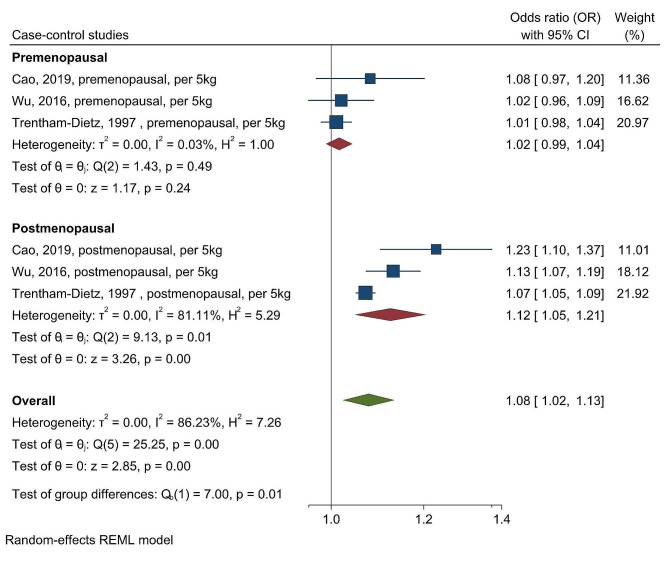
Forest plot for the association between per 5 kg weight change after age 18 and breast cancer risk in case-control studies, stratified by menopausal status

Additionally, we had similar findings in a separate meta-analysis for studies using continuous weight gain (per 5 kg) as a measurement. We observed a significant overall association for every 5 kg increase in weight and breast cancer incidence with an OR of 1.08 (95% CI, 1.02–1.13) (Fig. 3) with menopausal status being a source of heterogeneity (chi-squared test statistic of 7 and a *p*-value = 0.01; Fig. 3). The association between every 5 kg increase in weight and breast cancer incidence was significant only in postmenopausal women (OR = 1.12; 95%CI = 1.05–1.21; Fig. 3).

We did not observe any differences by country of study (chi-squared test statistic of 0.64 and a *p*-value = 0.42; Supplementary Fig. 4), or by hormone receptor status (chi-squared test statistic of 0.13 and a *p*-value = 0.72; Supplementary Fig. 5).

#### Cohort studies

We included 6 cohort studies with a total of 12 separate RRs (Supplementary Fig. 6). When comparing the highest versus the lowest categories of weight gain, we found a statistically significant overall association between weight gain after age 18 and breast cancer incidence, with an RR of 1.22 (95% CI, 1.09–1.36) (Supplementary Fig. 6).

**Fig. 4:**
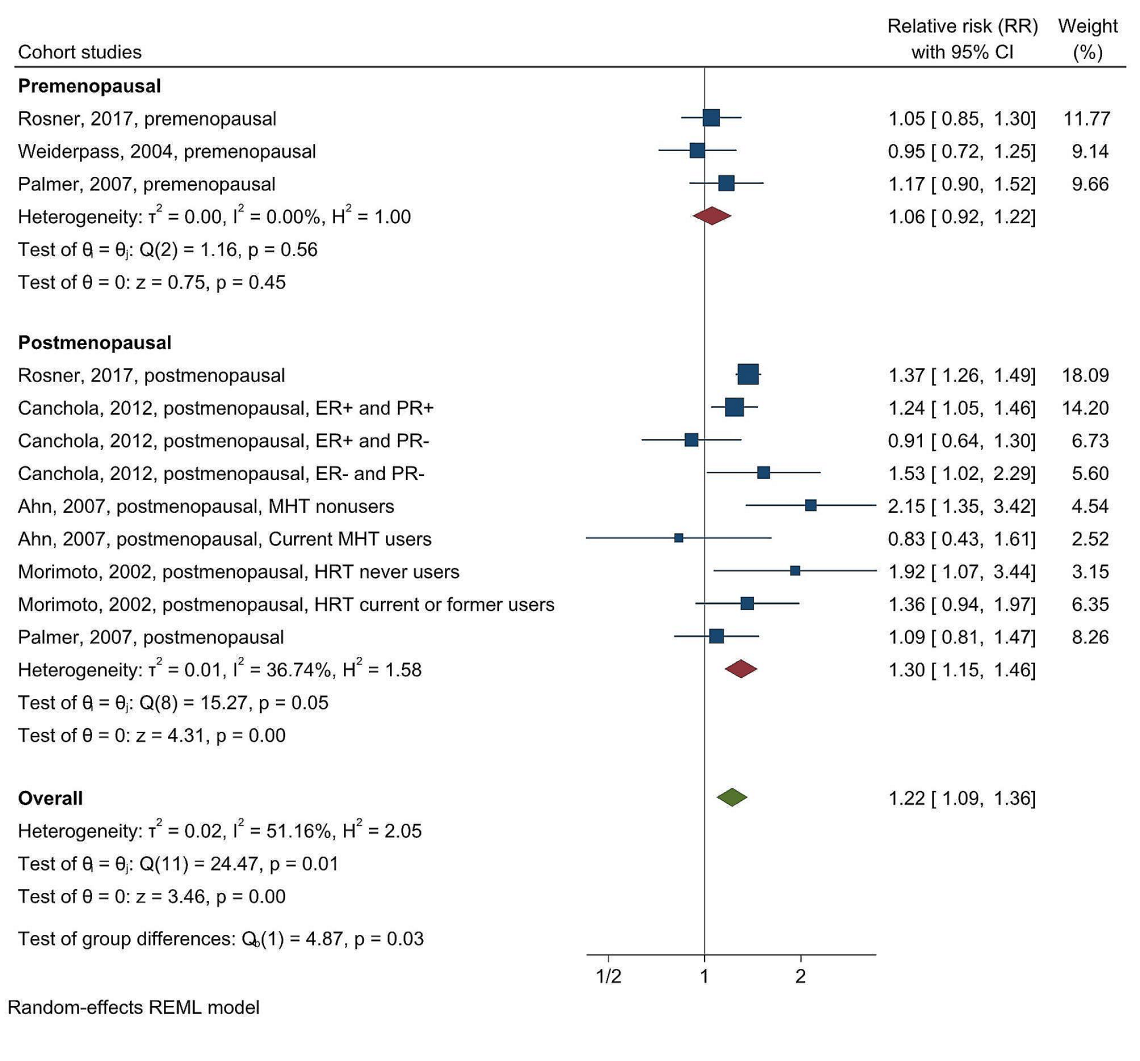
Forest plot for the association between weight gain after age 18 and breast cancer risk in cohort studies, stratified by menopausal status

After stratifying by menopausal status, we found that the association between weight gain after age 18 and breast cancer incidence was significant only in postmenopausal women (RR = 1.30; 95% CI = 1.15–1.46), not in premenopausal women (RR = 1.06; 95% CI = 0.92–1.22). The chi-squared test statistic of 4.87 and a *p*-value of 0.03 suggested that menopausal status was a significant source of heterogeneity (Fig. 4).

Excluding the cohort study by Rosner (2017), which reported HRs, did not change the overall pooled estimates in our meta-analysis. However, the chi-squared test statistic decreased to 1.92 with a *p*-value of 0.17, indicating that menopausal status was no longer a significant source of heterogeneity (Supplementary Fig. 7). Unfortunately, due to the limited number of available cohort studies and the lack of detailed information on certain variables, we were unable to conduct further investigations of potential sources of heterogeneity.

## Discussion

The present meta-analysis found that weight gain from age 18 was associated with an increased risk of postmenopausal breast cancer, but not with premenopausal breast cancer. Specifically, for every 5 kg increase in weight since age 18, there was a 12% rise in the risk of postmenopausal breast cancer. Notably, to our knowledge, this meta-analysis is the first to focus on weight gain since the age of 18 years as the starting point for early adulthood.

Our findings are consistent with a previous dose-response meta-analysis by Chan and colleagues, which reported that a 7% increase in the risk of postmenopausal breast cancer for every 5 kg weight gain in adulthood (RR, 1.07; 95% CI, 1.05–1.09) [10]. However, Chan’s analysis included studies with various early adulthood starting ages ranging from 15 to 25 years old [10]. In our analysis, we did not find any association between weight gain and hormone receptor status, which may be due to the limited number of studies that stratified by both hormone receptor status and menopausal status. However, Rosner and Chan found a significant positive association between adult weight gain and ER + PR + breast cancers in postmenopausal women, but not with ER + PR-/ER-PR- breast cancers [10, 22].

The precise mechanisms underlying the associations between long-term weight change, breast cancer, and the divergent effects of menopausal status remain poorly understood. However, some possible pathways include the influence of mammographic breast density [22, 33], which is a strong risk factor and intermediate marker of breast cancer risk [34]. For instance, in postmenopausal women, percent mammographic breast density may mediate up to 26% of the effect of childhood and adolescent somatotypes on breast cancer risk [35]. Initially, we anticipated an inverse association between long-term weight gain and premenopausal breast cancer overall, as suggested by Schoemaker et al.’s prospective pooled analysis results [5]. This expectation was based on two factors: (1) additional weight gain being associated with a reduction in mammographic density, and (2) substantial weight gain leading to obesity, which suppresses ovarian function, consequently reducing endogenous sex hormone exposure, particularly progesterone [5]. However, our results did not demonstrate an inverse association, possibly due to the inability to analyze breast cancer hormone receptor status. Transcriptomic analysis indicates that pathways involving proliferation, immune response, and inflammation may also play a role [36, 37]. For example, early-life adiposity has been linked to lower cellular proliferation pathways, including MYC target genes, in both estrogen receptor positive and negative breast tumors [37]. Further research is needed to understand the biological mechanisms underlying the association of adiposity change across the lifespan and breast cancer risk [38].

Recently, Mendelian randomization studies have provided additional insights into this complex relationship, suggesting that observational studies based on a single measurement may underestimate the magnitude of the association [39]. A recent review (Fang et al., 2021) summarized current evidence from Mendelian randomization studies, shedding light on the complex relationship between adiposity and different types of cancers, and providing further insight into the causality of the inverse association of early life adiposity with breast cancer [39].

Our study has several limitations. First, there may be a potential recall bias for self-reported weight during early adulthood [11]. However, previous studies have reported strong correlations between self-reported and measured weight and BMI, typically ranging between 0.87 and 0.92 [11, 40]. Second, although we carefully synthesized the data in this study, the results should be interpreted with caution due to the limitations of meta-analysis, which can introduce potential heterogeneities, including differences in exposure measurements, outcome reporting, and modeling in each included study. Further individual participant data (IPD) meta-analyses may improve data quality, and pooled analyses can also address these limitations [41]. A large pooled analysis of the participant-level data showed consistent results with our study [41]. Finally, we were unable to evaluate race as a potential source of heterogeneity, and we did not have sufficient data to assess the impact of hormone replacement therapy (HRT) use in postmenopausal women for this meta-analysis. A previous dose-response meta-analysis examining 10 obesity-related cancers, including breast cancer, found that each 5 kg increase in adult weight gain is associated with a statistically significant 11% increase in risk for postmenopausal breast cancer among individuals who do not use HRT [42]. These limitations underscore the need for further research to comprehensively understand the influence of factors between weight change and breast cancer risk.

## Conclusion

In conclusion, weight gain from age 18 is associated with an increased risk of breast cancer among postmenopausal women. This finding highlights the urgent need to enhance weight control from early adulthood to reduce a woman’s risk of postmenopausal breast cancer.

### Electronic supplementary material

Below is the link to the electronic supplementary material.

Supplementary Material 1

Supplementary Material 2

## Data Availability

The datasets used and/or analysed during the current study are available from the Dr. Han on reasonable request.

## Abbreviations

BMI: Body Mass Index
CI: Confidence Interval
HR: Hazard Ratio
NOS: Newcastle-Ottawa Scale
OR: Odds Ratio
RE: Risk Estimate
RR: Relative Risk
