# Supplementary material for: How does weight gain since the age of 18 years affect breast cancer risk in later life? A meta-analysis
Source: Breast Cancer Res. 2024 Mar 7;26:39. doi: 10.1186/s13058-024-01804-x (PMC10921610; doi:10.1186/s13058-024-01804-x)
Supplement: Supplementary file 1 — Supplementary Material 1 [file 13058_2024_1804_MOESM1_ESM.docx]

**Supplementary Tables and Figures**

**Title:** **How does weight gain since the age of 18 years affect breast cancer risk in later life?** **A meta-analysis**

**Supplementary Table 1. The Newcastle-Ottawa Scale (NOS) assessing the quality of case-control studies.**

| **Author** | **Year** | **Selection** | | | | **Comparability** | **Exposure** | | | **Total score** |
| --- | --- | --- | --- | --- | --- | --- | --- | --- | --- | --- |
|  |  | **Q1** | **Q2** | **Q3** | **Q4** | **Q1** | **Q1** | **Q2** | **Q3** |  |
| Cao | 2019 | ✰ | ✰ | ✰ | ✰ | ✰✰ | - | ✰ | - | 7 |
| Wu | 2016 | - | ✰ | ✰ | - | ✰✰ | - | ✰ | - | 5 |
| Iqbal | 2015 | - | ✰ | ✰ | ✰ | ✰✰ | - | ✰ | - | 6 |
| Robinson | 2014 | - | ✰ | ✰ | - | ✰✰ | - | ✰ | - | 5 |
| Kawai | 2014 | ✰ | ✰ | ✰ | - | ✰✰ | - | ✰ | - | 6 |
| Wenten | 2002 | - | ✰ | ✰ | - | ✰✰ | - | ✰ | - | 5 |
| Li | 2000 | - | ✰ | ✰ | ✰ | ✰✰ | - | ✰ | - | 6 |
| Magnusson | 1998 | ✰ | ✰ | ✰ | ✰ | ✰✰ | - | ✰ | - | 7 |
| Trentham-Dietz | 1997 | - | ✰ | ✰ | ✰ | ✰✰ | ✰ | ✰ | - | 7 |
| Taioli | 1995 | ✰ | ✰ | - | ✰ | ✰✰ | - | ✰ | - | 6 |
| Folsom | 1990 | ✰ | ✰ | ✰ | ✰ | ✰ | - | ✰ | - | 7 |

**Supplementary Table 2. The** **Newcastle-Ottawa Scale (NOS) assessing the quality of prospective cohort study studies.**

| **Author** | **Year** | **Selection** | | | | **Comparability** | **Outcome** | | | **Total score** |
| --- | --- | --- | --- | --- | --- | --- | --- | --- | --- | --- |
|  |  | **Q1** | **Q2** | **Q3** | **Q4** | **Q1** | **Q1** | **Q2** | **Q3** |  |
| Rosner | 2017 | - | ✰ | - | ✰ | ✰✰ | ✰ | ✰ | - | 6 |
| Canchola | 2012 | - | ✰ | - | ✰ | ✰✰ | ✰ | ✰ | ✰ | 7 |
| Ahn | 2007 | - | ✰ | - | ✰ | ✰✰ | ✰ | ✰ | - | 6 |
| Palmer | 2007 | - | ✰ | - | ✰ | ✰✰ | ✰ | ✰ | - | 6 |
| Weiderpass | 2004 | ✰ | ✰ | - | ✰ | ✰✰ | ✰ | ✰ | ✰ | 8 |
| Morimoto | 2002 | ✰ | ✰ | - | - | ✰✰ | ✰ | ✰ | ✰ | 7 |

**Supplementary Figure 1. Funnel plot of the risk estimates for the association between weight gain after age 18 and breast cancer risk in case-control studies.**

**Supplementary Figure 2. Funnel plot of the risk estimates for the association between weight gain after age 18 and breast cancer risk in cohort studies.**

**Supplementary Figure 3. Forest plot for the association between weight gain after age 18 and breast cancer risk in case-control studies.**

**Supplementary Figure 4. Forest plot for the association between weight gain after age 18 and breast cancer risk** **in case-control studies, stratified by country where the study was conducted.**

**Supplementary Figure 5. Forest plot for the** **association between weight gain after age 18 and breast cancer risk in case-control studies, stratified by hormone receptor status.**

**Supplementary Figure 6. Forest plot for the association between weight gain after age 18 and breast cancer risk** **in cohort studies.**

**Supplementary Figure 7.** Forest plot for the association between weight gain after age 18 and breast cancer risk in cohort studies (excluding Rosner 2017), stratified by menopausal status.
