# Supplementary material for: How does weight gain since the age of 18 years affect breast cancer risk in later life? A meta-analysis
Source: Breast Cancer Res. 2024 Mar 7;26:39. doi: 10.1186/s13058-024-01804-x (PMC10921610; doi:10.1186/s13058-024-01804-x)
Supplement: Supplementary file 2 — Supplementary Material 2 [file 13058_2024_1804_MOESM2_ESM.docx]

**Supplement.** **Full Search Strategies**

**Title:** **How does weight gain since the age of 18 years affect breast cancer risk in later life?** **A meta-analysis**

**Methods:**

The published literature was searched using strategies designed by a medical librarian for the concepts of weight gain or weight change, breast density, and breast cancer. These strategies were created using a combination of controlled vocabulary terms and keywords, and were executed in Medline (Ovid) 1946, Embase.com 1947-, Scopus 1823-, Cochrane Library (including CENTRAL), and Clinicaltrials.gov. Results were limited to English using database-supplied filters. A filter was also used to exclude animal-only studies from Ovid-Medline and Embase.^1^ The search was initially completed in February 2021 and was executed again in March 2022 and in June 2022. Full search strategies are provided.

**Results:**

The initial search completed in 2021 retrieved 7,653 results. These results were exported to Endnote software. 56 citations from ClinicalTrials.gov were retrieved and added to an Excel file library. After removing duplicates 4,368 unique citations remained for analysis. The updated search in March 2022 retrieved 611 results. From this search 319 duplicates were identified and removed, leaving 292 citations. The search was updated again in June 2022 retrieving 65 unique citations after removing duplicates. Between the initial and updated searches a total of 4,725 unique results remained for analysis.

Note: You may not need to include this citation for excluding animal studies. It is a fairly common filter and this is not its original creator (this site just does a good job of explaining the rationale for how the filter is designed).

1. McGill Library. “Advanced tools for building the search: Search filters and hedges.” Accessed February 19, 2021: <https://libraryguides.mcgill.ca/knowledge-syntheses/search-tools>

**Endnote Library Stats:**

Total number of retrieved: 7,653

Number of duplicates identified: 3340

Number of unique citations: 4368 (4312 from Endnote + 56 ClinicalTrials.gov results)

**Updated Search Citation Library Stats** **March, 2022:**

Total number retrieved: 611

Number of duplicates identified: 319 total (276 duplicates from updated search; 43 duplicates between original search and updated search)

Number of unique citations: 292

Total number of unique citations (original and updated search): 4660

**Updated Search Citation Library Stats, June 2022:**

Total number retrieved: 213

Number of duplicates identified: 148 total (102 duplicates from within updated search results; 46 duplicates between this search and the March 2022 search)

Number of unique citations: 65

**Total number of unique citations (original and two updated searches): 4,725**

**Please note:** The project librarian (Angela Hardi) should be included as an author on the systematic review manuscript if the search methods statement above or the search strategies listed below are included in the manuscript.

**Complete Search Strategies:**

**Embase.com**

= 3,670 results on 2/19/2020; Limited to English; Human filter used

**Updated search March 2022:** (results limited to 2021-present): 253 results on 3/17/2022

**Updated Search June 2022:** (results limited to 2022-present): 91 results on 6/3/2022

('body weight gain'/de OR 'weight trajectory (body weight)'/de OR (weight OR adiposity OR ‘body mass’ OR ‘BMI’) NEAR/2 (gain* OR change* OR trajector* OR increase*)) AND ('breast density'/exp OR 'breast cancer'/exp OR ((breast OR mammary OR mammographic) NEAR/2 (densit*)) OR ((breast OR mammary) NEAR/2 (cancer* OR neoplasm* OR carcinoma* OR ‘malignant tumor*’ OR ‘malignant tumour*’))) NOT ([animals]/lim NOT [humans]/lim) AND [english]/lim

**Ovid-Medline All**

= 1553 results on 2/19/2021; Limited to English; Human filter used

**Updated search March 2022:** (results limited to 2021-present): 150 results on 3/17/2022

**Updated search June 2022:** (results limited to 2022-present): 59 results on 6/3/2022

(Weight Gain/ OR Body-Weight Trajectory/ OR ((weight OR adiposity OR "body mass" OR "BMI") adj2 (gain* OR change* OR trajector* OR increase*)).mp.) AND ((Breast Density/ OR ((breast OR mammary OR mammographic) adj2 (densit*)).mp.) OR (exp Breast Neoplasms/ OR ((breast OR mammary) adj2 (cancer* OR neoplasm* OR carcinoma* OR "malignant tumor*" OR "malignant tumour*")).mp.)) NOT (Animals/ NOT (Animals/ AND Humans/))

**Scopus**

=1941 results on 2/19/2021; Limited to English; books and book chapters excluded

**Updated search March 2022:** (results limited to 2021-present): 174 results on 3/17/2022

**Updated search June 2022:** (results limited to 2022-present): 49 results on 6/3/2022

TITLE-ABS((weight W/2 gain*) OR (weight W/2 change*) OR (weight W/2 trajector*) OR (weight W/2 increase*) OR (adiposity W/2 gain*) OR (adiposity W/2 change*) OR (adiposity W/2 trajector*) OR (adiposity W/2 increase*) OR (“body mass” W/2 gain*) OR (“body mass” W/2 change*) OR (“body mass” W/2 trajector*) OR (“body mass” W/2 increase*) OR (“BMI” W/2 gain*) OR (“BMI” W/2 change*) OR (“BMI” W/2 trajector*) OR (“BMI” W/2 increase*)) AND (TITLE-ABS((breast W/2 densit*) OR (mammary W/2 densit*) OR (mammographic W/2 densit*)) OR TITLE-ABS((breast W/2 cancer*) OR (breast W/2 neoplasm*) OR (breast W/2 carcinoma*) OR (breast W/2 “malignant tumor*”) OR (breast W/2 “malignant tumour*”) OR (mammary W/2 cancer*) OR (mammary W/2 neoplasm*) OR (mammary W/2 carcinoma*) OR (mammary W/2 “malignant tumor*”) OR (mammary W/2 “malignant tumour*”))) ( EXCLUDE ( DOCTYPE , "ch" ) OR EXCLUDE ( DOCTYPE , "bk" ) ) AND ( LIMIT-TO ( LANGUAGE , "English") )

**Cochrane Library**

= 489 results on 2/19/2021 (3 Cochrane reviews, 485 CENTRAL Trials, and 1 Editorial. The editorial was not added to the Endnote library)

**Updated Search March 2022:** (results limited to 2021-present): 34 CENTRAL Trials

**Updated Search June 2022:** (results limited to 2022-present): 11 CENTRAL Trials

ID Search Hits

#1 MeSH descriptor: [Weight Gain] this term only 2536

#2 MeSH descriptor: [Body-Weight Trajectory] explode all trees 10

#3 ((weight OR adiposity OR "body mass" OR "BMI") NEAR/2 (gain* OR change* OR trajector* OR increase*)) :ti,ab,kw 21884

#4 #1 OR #2 OR #3 21884

#5 MeSH descriptor: [Breast Density] explode all trees 32

#6 ((breast OR mammary OR mammographic) NEAR/2 (densit*)) :ti,ab,kw 534

#7 #5 OR #6 534

#8 MeSH descriptor: [Breast Neoplasms] explode all trees 13211

#9 ((breast OR mammary) NEAR/2 (cancer* OR neoplasm* OR carcinoma* OR "malignant tumor*" OR "malignant tumour*")):ti,ab,kw 37070

#10 #8 OR #9 37083

#11 #7 OR #10 37202

#12 #11 AND #4 489

**ClinicalTrials.gov**

= 80 results on 2/22/2021 (56 remained after removing duplicates from the database search)

**Updated search June 2022:** 3 results (results limited to study start date 1/1/2022-6/3/2022)

EXPAND[Concept] ( ( "weight change" OR "weight gain" OR "weight trajectory" OR "weight increase" OR "body mass gain" OR "body mass increase" OR "body mass change" OR "adiposity change" OR "adiposity increase" OR "adiposity trajectory" ) AND AREA[ConditionSearch] ( "breast cancer" OR "breast carcinoma" OR "breast neoplasm" OR "breast density" OR "mammary density" OR "mammographic density" ) )
